# Research on the Method of Depth-Sensing Optical System Based on Multi-Layer Interface Reflection

**DOI:** 10.3390/s24227228

**Published:** 2024-11-12

**Authors:** Chen Yu, Ying Liu, Linhan Li, Guangpeng Zhou, Boshi Dang, Jie Du, Junlin Ma, Site Zhang

**Affiliations:** 1 Changchun Institute of Optics, Fine Mechanics and Physics, Chinese Academy of Sciences, Changchun 130033, China; yuchen21@mails.ucas.ac.cn (C.Y.); lilinhan22@mails.ucas.ac.cn (L.L.); zhouguangpeng19@mails.ucas.ac.cn (G.Z.); dangboshi@163.com (B.D.); dj_ciomp@163.com (J.D.); michael8446@126.com (J.M.); zhangsite@ciomp.ac.cn (S.Z.); 2 University of Chinese Academy of Sciences, Beijing 100049, China

**Keywords:** transparent biological samples, microscopy, depth-sensing system, semi-annular diaphragm

## Abstract

In this paper, a depth-sensing method employing active irradiation of a semi-annular beam is proposed for observing the multi-layered reflective surfaces of transparent samples with higher resolutions and lower interference. To obtain the focusing resolution of the semi-annular aperture diaphragm system, a model for computing the diffracted optical energy distribution of an asymmetric aperture diaphragm is constructed, and mathematical formulas are deduced for determining the system resolution based on the position of the first dark ring of the amplitude distribution. Optical simulations were performed under specific conditions; the lateral resolution $\delta_{r}$ of the depth-sensing system was determined to be 0.68 μm, and the focusing accuracy $\delta_{z}$ was determined to be 0.60 μm. An experimental platform was established under the same conditions, and the results were in accord with those of the simulation results, which validated the correctness of the formula for calculating the amplitude distribution of the diffracted light from the asymmetric aperture diaphragm.

## 1. Introduction

Optical microscopes are essential tools across various scientific and technological domains, prominently utilized in bioengineering, flow cytometry, gene sequencing, pathological diagnosis, semiconductor processing, wafer inspection, photolithography, materials science, and other disciplines. Autofocus technology plays a pivotal role in enhancing the speed and accuracy of microscope focusing. Defocusing of the optical system significantly diminishes the high-frequency spatial information of imaging [1]. Kujoory, Mayall, and Mendelsohn et al. conducted a comparative analysis between manual focusing microscopes and those assisted by autofocus technology employing the same algorithm. Their findings revealed that autofocus accuracy surpasses manual focusing by fourfold [2,3]. In pursuit of optimizing microscope imaging quality and efficiency, numerous intricate autofocus methodologies have been proposed. However, prevalent autofocus techniques in microscopes are currently categorized into two main types: active and passive. Passive autofocus relies on image sharpness recognition, offering superior reliability and robustness in obtaining a clear image at the precise focus position [4,5,6]. Nonetheless, this approach is time-consuming, necessitating prolonged exposure times for each imaging session, coupled with extensive image sharpness analysis [7,8,9,10]. For instance, high-throughput DNA sequencing processes demand rapid data processing with mere tens of milliseconds between imaging neighboring DNA sample regions [11,12,13,14]. Conversely, active autofocus gauges the distance between the sample and the objective lens by emitting a laser beam of a specific wavelength, subsequently regulating a stepper motor to achieve focusing based on the functional relationship between the image center of mass position and the degree of defocus [15,16,17,18]. It exhibits swifter response rates and heightened focusing precision, thereby finding extensive applications in industrial sectors necessitating real-time automated optical inspection and machining [19]. Nevertheless, none of the aforementioned methods facilitate automatic focusing on multiple reflective surfaces of complex structural samples. When imaging a solitary reflective surface, other reflective surfaces may concurrently project onto the CCD, inducing interference, thereby leading to focusing errors due to the incapacity to discern multiple reflective surfaces. Furthermore, traditional active autofocus microscopes suffer from low resolutions and lack a direct resolution calculation method, rendering them unsuitable for the high-resolution imaging of transparent biological samples featuring three-dimensional structures.

This paper investigates and designs a novel depth-sensing microscope system to address the aforementioned issues. It employs a semi-annular aperture diaphragm to enable real-time imaging of multi-layer reflective surfaces in samples. Additionally, it achieves rapid focusing of the samples’ complex three-dimensional structures by formulating linear equations for various reflective surfaces and out-of-focus quantities. The optical simulation confirms that this system offers superior resolution compared to traditional active semi-circular aperture diaphragm autofocus microscope systems. Theoretical analysis in this paper extends to deriving the beam-shaping characteristics of asymmetric aperture diaphragms through Fraunhofer diffraction. This supplements the traditional Fresnel diffraction formula, which is limited to symmetric aperture diaphragms. Currently, there are relatively few related methods at home and abroad for calculating the field intensity distribution of asymmetric aperture diaphragms, and the mathematical model for autofocus resolution has not been fully established. Therefore, the numerical calculation method proposed in this paper holds significant importance. The proposed mathematical calculation method for determining the energy distribution of irregular aperture diaphragms is validated through an experimental optical platform.

## 2. Principle of Fast Depth-Sensing Microscope

**Figure 1:**
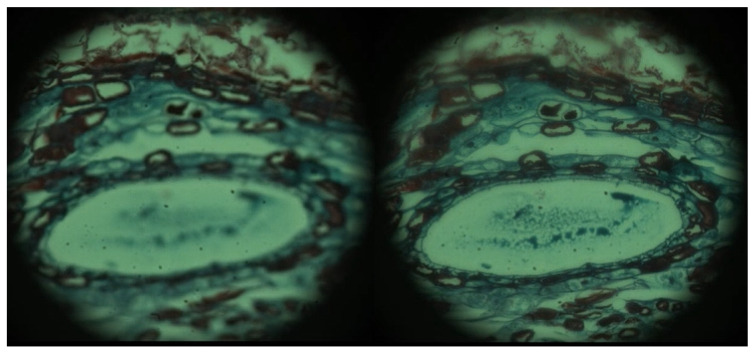
Focus map of cells at different levels of the pine stem.

Figure 1 displays a picture of pine stem cells under a microscope. It can be observed from this figure that, due to the stacking of cells, the sample shows different layers. When a certain layer of cells is positioned in the focal plane, the other layers will become blurred due to being out-of-focus. Consequently, single-layer focusing has certain limitations and cannot satisfy the requirements for observation.

**Figure 2:**
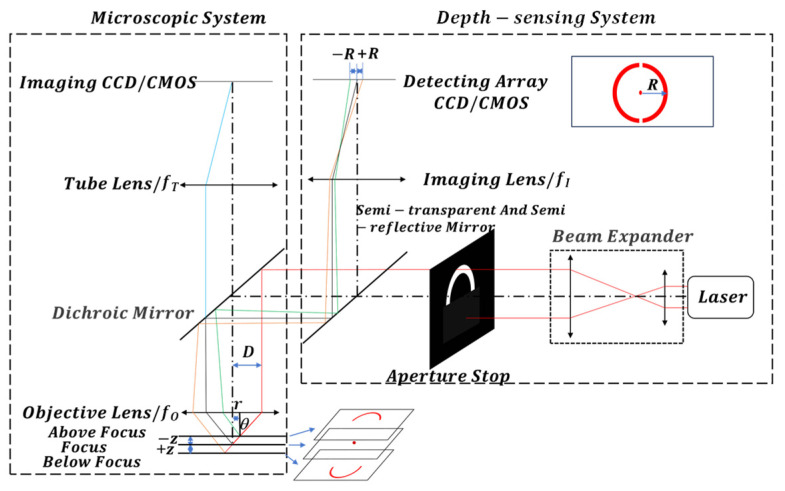
Schematic diagram of the principle of fast depth-sensing with multi-layer transparent surface.

Usually, biological samples exhibit multiple reflective surfaces, such as the upper coverslip, microfluidic cavity channel, and the lower slide. Within the cavity channel, the detection targets are randomly distributed across both the upper and lower surfaces, necessitating the focus on multiple surfaces of the sample. Figure 2 illustrates the principle of rapid depth-sensing on multiple transparent surfaces. The laser light passes through a beam expander, transmitting the expanded beam onto the semi-annular aperture diaphragm’s surface, creating a semi-annular parallel beam. This beam then traverses a beam splitter mirror to reach the dichroic mirror within the microscope system. Subsequently, it is reflected into the micro-objective lens, reaching the transparent sample. As the light reflects off different surfaces of the transparent sample, it passes back through the dichroic mirror and beam splitter. The imaging mirror converges the light spot onto the CCD/CMOS for calculation. Based on the graphical distribution of the CCD/CMOS surface array, the number of layers of the transparent sample and the imaging position of the microscope system are determined. In this schematic, the transparent sample is configured with three reflective surfaces, and the focus is set on the middle reflective surface to facilitate rapid identification of different surfaces through the position of the center of mass captured by the CCD sensor. To ensure that the focus position of the microscope is the same as the focus position of the depth-sensing device, the achromatic objective lens is chosen here to eliminate the error caused by the difference between the wavelength of the laser and the wavelength of the light illuminated by the microscope. Notably, key parameters determining the performance of the entire depth-sensing system include focusing accuracy, focusing range, and image recognition effectiveness.

In cases of a certain surface of microscopic observation of the sample wherein the microscope stage is moved to level the focal plane of the objective lens, the observation surface is focused on the CCD/CMOS center imaging. When the stage is moved, causing the observation surface to be out of focus, the imaging spot increases for the off-center semi-annular, and the spot is oriented in a different direction when positive and negative are out of focus. By tracing the direction of the half-ring deviation and the size of the outer diameter, the direction and size of the out-of-focus can be known so as to realize the depth-sensing of the observation surface. In addition, the other layers of the sample have different distances relative to the objective lens, and the CCD/CMOS image is a different half-ring spot, so the same tracing method can be used for real-time focusing observation of different layers of the sample.

## 3. Calculation and Analysis of Depth-Sensing System Performance Parameters

The depth-sensing system determines the degree of out-of-focusedness by analyzing changes in the graphical displacement on the CCD/CMOS. To resolve the interlayer spacing of various reflective surfaces in transparent samples, establishing the correlation between the half-ring graphic and the degree of out-of-focusedness on the CCD/CMOS array of the depth-sensing system is essential. Figure 2 depicts a system wherein we disregard the thickness of the half-ring and focus solely on the outer radius of the spot and the degree of out-of-focusedness. This system, depicted in Figure 2, consists of the microscope objective lens with focal length ($f_{o}$), imaging lens with focal length ($f_{I}$), an incident laser beam with a half aperture (*D*), and laser irradiation at an angle (θ) to the sample’s reflective surface. If a reflective surface of the sample is *z* distance away from the objective lens’s focal plane, it is imaged in an out-of-focus position, resulting in a spot with radius *r*. *r* is the distance between the origin and the centroid of the spot. This spot corresponds to an image on the surface array CCD/CMOS with a radius *R*. *R* is the distance between the origin and the centroid of the spot. The laser beam is directed onto the sample’s reflective surface at an angle θ. In the triangle formed by the incident laser, the vertical optical axis of the microscope, and the objective lens’s focal plane, the following relation holds:

(1) $$\frac{z}{f_{0}}=\frac{r}{D}$$

The detector imaging spot radius satisfies the imaging relationship with the spot radius on the out-of-focus plane:

(2) $$\frac{R}{r}=\frac{f_{I}}{f_{O}}$$

From the object–image relationship, the radius of the spot on the detecting surface-array CCD/CMOS is as follows:

(3) $$R=z\cdot \frac{f_{I}}{f_{O}}\cdot \tan\theta$$

Equation (3) demonstrates a clear linear correlation between the semi-annular radius observed by the sensor and the extent of out-of-focusedness. Through this direct relationship, the degree of out-of-focusedness can be determined by tracking changes in the center of mass coordinates of the image. The depth-sensing system’s focusing range is influenced by both the outer diameter of the annular spot and the area of the detector target surface, as defined in Equation (3). With a fixed detector target surface size, an increase in the focal length of the imaging lens results in a larger outer spot diameter, thus reducing the focusing range. In essence, the focusing range is directly proportional to the size of the detector target surface and inversely proportional to the focal length of the imaging lens. In this paper, the depth-sensing system features a focusing range of ±200 μm. This range interval not only guarantees that the spot is distinctly displayed on the detector with clear imaging but also that it surpasses the vertical positioning error of the automatic stage in most microscope systems, thereby effectively fulfilling the depth-sensing function of the microscope system.

Deriving Equation (3), the expression becomes

(4) $$\delta_{R}=\frac{f_{I}}{f_{O}}\cdot \tan\theta \cdot \delta_{z}$$

$\delta_{R}$ in Equation (4) represents the imaging resolution of the depth-sensing system. When the imaging spot is smaller than the image element size of the CCD/CMOS, its size is determined by the image element size; conversely, it is determined by the size of the imaging spot of the depth-sensing system. $\delta_{z}$ represents the focusing accuracy of the depth-sensing system, indicating its ability to capture clear images of reflective surfaces of transparent samples. When *θ*, $f_{O}$, and $\delta_{R}$ are constant, $\delta_{z}$ and $f_{I}$ exhibit inverse proportionality, meaning that higher focal lengths of the imaging lens correspond to increased focusing accuracy. This relationship is evident through the microscope imaging process:

(5) $$\delta_{R}=\frac{f_{I}}{f_{O}}\cdot \delta_{r}$$

where $\delta_{r}$ is the microscope’s ability to resolve two object points on a given reflective surface, so $\delta_{z}$ can be deformed as

(6) $$\delta_{z}=\frac{\delta_{r}}{\tan\theta }$$

Equation (6) demonstrates that for a fixed angle of incident light, $\delta_{z}$ and $\delta_{r}$ exhibit a linear relationship, allowing the calculation of the depth-sensing system’s focusing accuracy $\delta_{z}$ based on the value of $\delta_{r}$.

The identification of neighboring surfaces of the sample should satisfy the Rayleigh Criterion, so the resolution between the sample levels determines the accuracy of the depth-sensing system and should also be larger than the detector’s pixel size. In the aforementioned analysis of the system’s performance parameters, the thickness of the semi-annular spot was disregarded. To achieve a precise determination of the microscope system resolution, it is imperative to conduct a detailed analysis of the energy of the diffracted light field beyond the semi-annular aperture diaphragm.

## 4. Calculation of Far-Field Diffracted Optical Energy Distribution from a Semi-Annular Aperture Diaphragm

The imaging quality of the detection surface array CCD/CMOS determines the focus recognition. The presence of a semi-annular aperture diaphragm introduces a significant diffraction effect to the system, potentially causing noise and diminishing the energy of the central bright spot. However, to date, no relevant article has derived the formula for the far-field diffraction light intensity distribution of the asymmetric aperture diaphragm. In this study, leveraging the Fraunhofer diffraction formula, we establish a model for calculating far-field amplitude distribution using approximate and cumulative methods. We compute the optical field energy distribution for both semi-circular and semi-annular aperture diaphragms and determine the resolution of the depth-sensing microscope system based on the position of the amplitude zero point.

**Figure 3:**
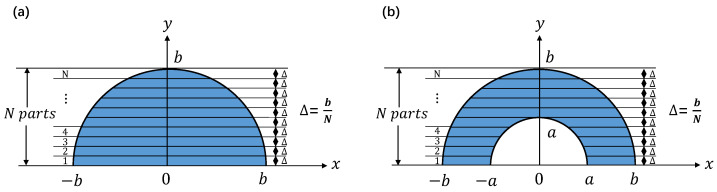
Computational modeling of diffracted light fields. (**a**) is a schematic diagram of the computational model for the diffracted light field distribution of a semi-circular aperture diaphragm. (**b**) is a schematic diagram of the computational model for the diffracted light field distribution of a semi-annular aperture diaphragm.

The calculation method is shown in Figure 3. For a semi-circular aperture diaphragm with radius length *b*, make its bottom edge horizontal so it may serve as the *x*-axis, and let the *y*-axis be vertical. The *y*-axis coincides with the symmetry axis of the aperture diaphragm. Along the *x*-axis, the diaphragm is cut into *N* parts, so the whole semi-circular aperture diaphragm is divided into *N* approximate rectangles with width $\frac{b}{N}$. When viewed along the *y*-axis, the length of the approximate rectangles decreases with the shortening of the *x*-axis interval, while the width remains constant at $\frac{b}{N}$.

Therefore, the diffracted intensity distribution of the semi-circular aperture diaphragm can be equated to the accumulation of the diffracted intensity distribution of *N* approximate rectangular aperture diaphragms. The mathematical derivation process is as follows:

The following is according to the Fraunhofer diffraction formula:

(7) $$E\left(x,y\right)=\frac{e^{jkf}}{j\lambda f}\cdot e^{\frac{jk}{2f}(x^{2}+y^{2})}\cdot \iint t(x_{0},y_{0})\cdot e^{-j2\pi \left(\frac{x}{\lambda f}x_{0}+\frac{y}{\lambda f}y_{0}\right)}d_{x_{0}}d_{y_{0}}$$

Let the radius of the diaphragm be *b*, the focal length of the objective lens be $f_{o}$, the wavelength of the incident light be *λ*, and the diaphragm amplitude transmittance be *t*($x_{0}$, $y_{0}$). According to Equation (7), it can be seen that the light beam through the semi-annular diaphragm diffraction occurs, and the objective lens focuses on the sample level of the amplitude of a light *E*(*x*, *y*) situation, which is

(8) $$\begin{array}{l} E\left(x,y\right)=\frac{e^{jkf}}{j\lambda f}\cdot e^{\frac{jk}{2f}\left(x^{2}+y^{2}\right)}\cdot \left[\int_{-b}^{b}t_{x_{0}}\cdot e^{-j2\pi \left(\frac{x}{\lambda f}x_{0}\right)}d_{x_{0}}\right.\cdot \int_{0}^{\frac{b}{N}}t_{y_{0}}\cdot e^{-j2\pi \left(\frac{y}{\lambda f}y_{0}\right)}d_{y_{0}}+\\ \;\;\;\;\;\int_{-b+\frac{b}{N}}^{b-\frac{b}{N}}t_{x_{0}}\cdot e^{-j2\pi \left(\frac{x}{\lambda f}x_{0}\right)}d_{x_{0}}\cdot \int_{\frac{b}{N}}^{\frac{2b}{N}}t_{y_{0}}\cdot {\;e}^{-j2\pi \left(\frac{y}{\lambda f}y_{0}\right)}d_{y_{0}}+\cdots +\\ \;\;\;\;\;\int_{-\frac{b}{N}}^{\frac{b}{N}}t_{x_{0}}\cdot e^{-j2\pi \left(\frac{x}{\lambda f}x_{0}\right)}d_{x_{0}}\cdot \left.\int_{b-\frac{b}{N}}^{b}t_{y_{0}}\cdot e^{-j2\pi \left(\frac{y}{\lambda f}y_{0}\right)}d_{y_{0}}\right] \end{array}$$

It is known to be equally divided for the *y*-direction so that the value of the integral at different *y*-axis positions is certain while the phase is constantly increasing:

(9) $$\begin{array}{l} E\left(x,y\right)=\frac{e^{jkf}}{j\lambda f}\cdot e^{\frac{jk}{2f}\left(x^{2}+y^{2}\right)}\cdot \left\{\int_{0}^{\frac{b}{N}}t_{y_{0}}\cdot e^{-j2\pi \left(\frac{y}{\lambda f}y_{0}\right)}dy_{0}\cdot \left[\int_{-b}^{b}t_{x_{0}}\cdot e^{-j2\pi \left(\frac{x}{\lambda f}x_{0}\right)}dx_{0}\right.\right.+\\ \;\;\;\;\;\int_{-b+\frac{b}{N}}^{b-\frac{b}{N}}{t_{x_{0}}\cdot e}^{-j2\pi \left(\frac{x}{\lambda f}x_{0}\right)}{\cdot e}^{-j2\pi \left(\frac{y}{\lambda f}\cdot \frac{b}{N}\right)}dx_{0}+\cdots +\\ \;\;\;\;\;\left.\int_{-\frac{b}{N}}^{\frac{b}{N}}{t_{x_{0}}\cdot e}^{-j2\pi \left(\frac{x}{\lambda f}x_{0}\right)}\left.{\cdot e}^{-j2\pi \frac{y}{\lambda f}\left(b-\frac{b}{N}\right)}dx_{0}\right]\right\} \end{array}$$

Calculated from Equation (9):

(10) $$\begin{array}{l} E\left(x,y\right)=\frac{e^{jkf}}{j\lambda f}\cdot e^{\frac{jk}{2f}\left(x^{2}+y^{2}\right)}\cdot E_{0}\left(x_{0},y_{0}\right)\cdot \frac{\sin\left(\frac{ky}{f}\cdot \frac{b}{2N}\right)}{\frac{ky}{f}}\cdot \left[\frac{2\sin\left(\frac{kx}{f}\cdot b\right)}{\frac{kx}{f}}\right.+\\ \;\;\;\frac{2\sin\left[\frac{kx}{f}\cdot \left(b-\frac{b}{N}\right)\right]}{\frac{kx}{f}\cdot }{\cdot e}^{-j2\pi \left(\frac{y}{\lambda f}\cdot \frac{b}{N}\right)}+\cdots +\left.\frac{2\sin\left(\frac{ky}{f}\cdot \frac{b}{N}\right)}{\frac{kx}{f}}{\cdot e}^{-j2\pi \frac{y}{\lambda f}\left(1-\frac{1}{N}\right)b}\right] \end{array}$$

Simplify Equation (10) to obtain the final form:

(11) $$E\left(x,y\right)=A\cdot E_{0}\left(x_{0},y_{0}\right)\cdot \frac{2\sin\left(\frac{ky}{f}\cdot \frac{b}{2N}\right)}{\frac{ky}{f}}\cdot \underset{n=0}{\overset{N-1}{\sum }}{\frac{\sin\left[\frac{\mathrm{kx}}{f}\cdot \frac{(N-n)b}{N}\right]}{\frac{kx}{f}}\cdot e}^{-j2\pi \frac{y}{\lambda f}\cdot \frac{nb}{N}}$$

Equation (11) represents the distribution of optical amplitude following the field of a semi-circular aperture diaphragm, where A denotes the complex amplitude generated by an approximate rectangular aperture. The spot dark ring position is determined by the term $\frac{2\sin\left(\frac{ky}{f}\cdot \frac{b}{2N}\right)}{\frac{ky}{f}}\cdot \overset{N-1}{\underset{n=0}{\sum }}{\frac{\sin\left[\frac{\mathrm{kx}}{f}\cdot \frac{(N-n)b}{N}\right]}{\frac{kx}{f}}\cdot e}^{-j2\pi \frac{y}{\lambda f}\cdot \frac{nb}{N}}$ in Equation (11).

The identical method was applied to subdivide the centrally shaded semi-annular aperture diaphragm illustrated in Figure 3b, featuring an outer radius of length *b* and an inner radius of length *a*. The resulting semi-annular amplitude distribution was derived as Equation (12).

(12) $$\begin{array}{l} E\left(x,y\right)=A\cdot E_{0}\left(x_{0},y_{0}\right)\cdot \frac{2\sin\left(\frac{ky}{f}\cdot \frac{b}{2N}\right)}{\frac{ky}{f}}\cdot \left\{{\overset{N-1}{\underset{n=0}{\sum }}\frac{\sin\left[\frac{kx}{f}\cdot \frac{\left(N-n\right)b}{N}\right]}{\frac{kx}{f}}\cdot e}^{-j2\pi \frac{y}{\lambda f}\cdot \frac{nb}{N}}\right.-\\ \;\;\;\left.{\overset{\frac{aN}{b}-1}{\underset{n=0}{\sum }}\frac{\sin\left[\frac{kx}{f}\left(a-\frac{b}{N}\cdot n\right)\right]}{\frac{kx}{f}}\cdot e}^{-j2\pi \frac{y}{\lambda f}\cdot \frac{nb}{N}}\right\} \end{array}$$

When *a* = 0, the semi-circular aperture diaphragm’s shape aligns precisely with that of the semi-circular aperture diaphragm, rendering the expression in Equation (12) as $E\left(x,y\right)=A\cdot E_{0}\left(x_{0},y_{0}\right)\cdot \frac{2\sin\left(\frac{ky}{f}\cdot \frac{b}{2N}\right)}{\frac{ky}{f}}\cdot \overset{N-1}{\underset{n=0}{\sum }}{\frac{\sin\left[\frac{\mathrm{kx}}{f}\cdot \frac{(N-n)b}{N}\right]}{\frac{kx}{f}}\cdot e}^{-j2\pi \frac{y}{\lambda f}\cdot \frac{nb}{N}}$, identical to that of Equation (11). Consequently, this paper focuses on discussing and analyzing Equation (12), with Equation (11) considered a special case within Equation (12).

Morphing Equation (12) by replacing all of $\frac{a}{b}$ with *τ* yields Equation (13).

(13) $$\begin{array}{l} E\left(x,y\right)=A\cdot E_{0}\left(x_{0},y_{0}\right)\cdot \frac{2\sin\left(\frac{ky}{f}\cdot \frac{b}{2N}\right)}{\frac{ky}{f}}\cdot \left\{{\overset{N-1}{\underset{n=0}{\sum }}\frac{\sin\left[\frac{kx}{f}\cdot \frac{\left(N-n\right)b}{N}\right]}{\frac{kx}{f}}\cdot e}^{-j2\pi \frac{y}{\lambda f}\cdot \frac{nb}{N}}\right.-\\ \;\;\;\left.{\overset{\tau N-1}{\underset{n=0}{\sum }}\frac{\sin\left[\frac{kx}{f}\left(\frac{\tau N-n}{N}\right)b\right]}{\frac{kx}{f}}\cdot e}^{-j2\pi \frac{y}{\lambda f}\cdot \frac{nb}{N}}\right\} \end{array}$$

## 5. Simulation Calculation and Analysis

A discussion and analysis of Equation (12) shows that it can be divided into two cases depending on the value of *N:*

**Figure 4:**
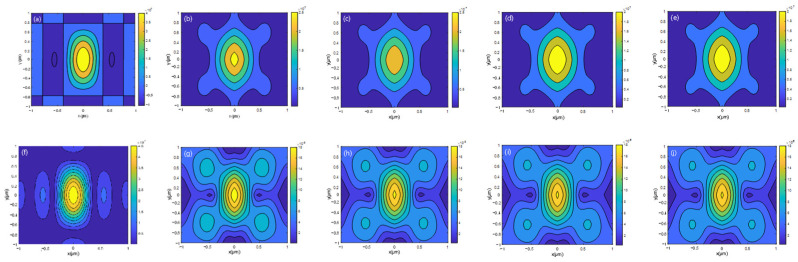
Far-field diffraction amplitude profiles of semi-circular and semi-annular aperture diaphragms at different *N* values. (**a**–**e**) are the far-field diffractograms of semi-circular aperture diaphragms obtained when *N* = 1, *N* = 10, *N* = 50, *N* = 100, and *N* = 1000, respectively. (**f**–**j**) are also the far-field diffractograms of semi-annular aperture diaphragms obtained at *N* = 1, *N* = 10, *N* = 50, *N* = 100, and *N* = 1000, respectively.

(1) When *N* = 1, Equation (12) simplifies to $E\left(x,y\right)=A\cdot E_{0}\left(x_{0},y_{0}\right)\cdot \frac{2\sin\left(\frac{ky}{f}\cdot \frac{b}{2}\right)}{\frac{ky}{f}}\cdot \frac{\sin\left(\frac{kx}{f}\cdot b\right)}{\frac{kx}{f}}$, indicating that the far-field diffraction amplitude distribution of the semi-annular aperture diaphragm is equivalent to that of the rectangular aperture diaphragm with a length of 2*b* and a width of *b*. However, under this condition, the results deviate more from the actual results.

(2) When *N* ≫ 1, the subdivided rectangle increasingly matches the shape of the semi-circular and semi-annular aperture diaphragms. To verify this, light field amplitude distribution curves were obtained at different *N* values: *N* = 1, 10, 50, 100, and 1000, respectively. The amplitude distribution curves are plotted according to Equations (11) and (12), as shown in Figure 4.

Figure 4 reveals that the amplitude curves nearly overlap when *N* = 100 and *N* = 1000, suggesting that Equations (11) and (12) serve as the far-field diffraction amplitude expressions for semi-circular and semi-annular aperture diaphragms when *N* ≥ 100.

**Figure 5:**
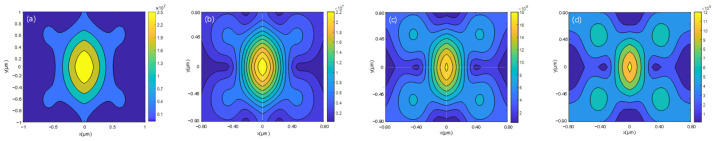
Amplitude distribution curve of half-ring aperture diaphragm. Plot of the far-field diffraction amplitude distribution from the semi-annular aperture diaphragm. (**a**–**d**) are three-dimensional plots of the amplitude distribution obtained at *τ* = 0, *τ* = 0.2, *τ* = 0.5, and *τ* = 0.7, respectively.

To simulate a general-purpose microscope, the outer radius of the semi-annular aperture diaphragm is set to *b* = 10 mm (matching the diameter of the through-hole of a conventional microscope), the focal length of the objective lens is *f* = 10 mm (corresponding to a 20× microscope objective lens), the laser wavelength is λ = 785 nm (to avoid interference with visible-light microscope imaging). For Equation (12), when *a* = *τb*, simulation plots of amplitude distribution at different values of *τ* are generated, and the results are depicted in Figure 5.

In Figure 5, it can be observed that when the wavelength of the laser is 785 nm, the curve gradually contracts inward as *τ* increases. The peak amplitude decreases continuously, and the imaging spot size diminishes. The horizontal axis of the graph represents the distance from the central point in the *x*-direction, the vertical axis indicates the distance from the central point in the *y*-direction, and different colors represent different amplitudes.

Regardless of the value of *τ*, it is observed that the first zero point of the amplitude along the *y*-axis of the spot is consistently approximately twice that along the *x*-axis. This means the radius of the first dark ring on the *y*-axis of the imaging spot is approximately twice that on the *x*-axis. Compared with the traditional circular aperture microscopy system, the *y*-axis direction resolution is no longer consistent with the *x*-axis direction resolution, and its resolution calculation no longer follows the traditional circular aperture resolution formula but is dominated by the *y*-axis direction resolution, and it can be seen in Figure 5 that the resolution of the semi-annular aperture diaphragm microscopy system is larger than the resolution of the semi-circular aperture diaphragm microscopy system. The variation of the coordinate values of the first amplitude zero point with *τ* in the *x*-axis and *y*-axis in Figure 5 is shown in Table 1.

As the term $\frac{2\sin\left(\frac{ky}{f}\cdot \frac{b}{2N}\right)}{\frac{ky}{f}}$ in Equation (12) is solely dependent on the variable *b*, the parameters in Table 1 are associated with *b*. Thus, recalculations were performed for *b* values of 5 mm and 15 mm, yielding the results presented in Table 2 and Table 3, respectively.

Comparing Table 1, Table 2 and Table 3 reveals that when the ring widths are held constant at 3 mm, corresponding to *b* = 5 mm for *τ* = 0.6, *b* = 10 mm for *τ* = 0.3, and *b* = 15 mm for *τ* = 0.2, the resolutions of their respective semi-annular aperture diaphragm microscopy systems vary and increase with the increment of *b* value. Furthermore, with a constant *τ* value and increasing *b* value, the resolution of the semi-annular aperture diaphragm microscope system continues to rise. However, when *b* remains constant and *τ* increases, although the system’s resolution gradually improves, the reduction in light transmission area results in a decrease in the primary amplitude of light, deteriorating spot imaging quality. Moreover, the secondary peak amplitude increases, leading to heightened noise. The system’s signal-to-noise ratio is solely influenced by the *τ* value, decreasing as *τ* increases.

**Figure 6:**
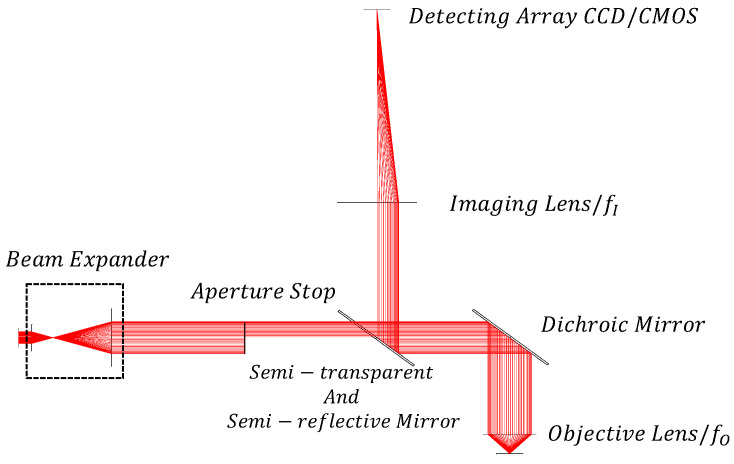
Structure of the optical path of the semi-annular aperture diaphragm depth-sensing system.

**Figure 7:**
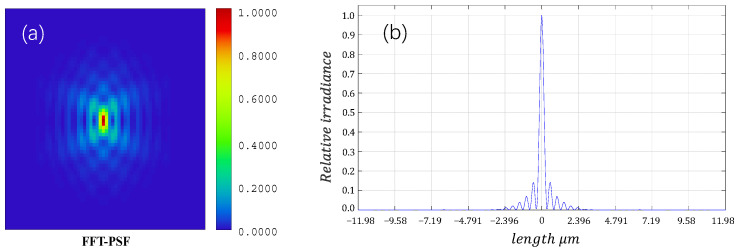
Schematic diagram of the distribution of far-field diffracted light field from a semi-annular aperture diaphragm. (**a**) is a light energy simulation diagram when focused. (**b**) is a simulation diagram of relative irradiance.

In consideration of imaging quality and resolution, the system opts for a stop diaphragm with an outer radius of 10 mm and an inner radius of 8 mm. Zemax 2019 software is employed to conduct optical simulations of the depth-sensing system, with the optical structure detailed as follows:

Figure 6 shows the schematic diagram of the optical path situation of the depth-sensing system during focusing, and its light energy distribution is shown in Figure 7.

In Figure 7, when the illumination is zero, the resolution of the *y*-axis of the Airy spot is approximately 0.68 μm, consistent with the findings of the aforementioned analysis. Therefore, with *b* = 10 mm and *a* = 8 mm, $\delta_{r}$ is 0.68 μm. By Equation (6), the $\delta_{z}$ of the depth-sensing system can be derived as 0.60 μm. This implies that the detector can effectively distinguish between the reflective surfaces of the confocal position and their neighboring surfaces when the distance between them is 0.60 μm. The diagram illustrating the detector point column is provided below.

**Figure 8:**
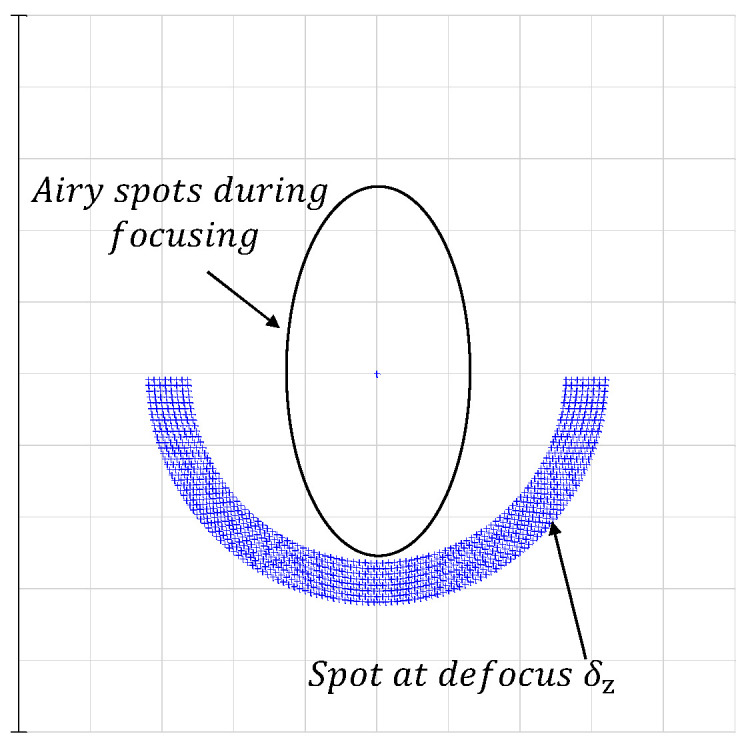
List of detector points for the reflecting surface at the confocal position and the reflecting surface at the defocused $\delta_{z}$ position.

In Figure 8, the geometric spot formed by the reflection surface located at the focal plane position is much smaller than the Airy spot size, so the Airy spot is the actual spot size at this time. At this point, the inner diameter of the semi-annular out-of-focus spot aligns with the Airy spot, which means these two reflective surfaces can be separated exactly.

The depth of field formula for infinity microscope objectives is given as follows:

(14) $$d_{tot}=\frac{\lambda \cdot n}{{NA}^{2}}+\frac{n}{M\cdot NA}e$$

here $d_{tot}$ represents the depth of field, *λ* is the operational wavelength of the microscope, *n* refers to the medium between the coverslip and the lens element in front of the objective lens, *M* indicates the transverse magnification of the micro-objective lens, and *e* signifies the minimum resolvable distance by the detecting surface array CCD/CMOS. When considering a micro-objective with a transverse magnification *M* of 20× and a numerical aperture *NA* of 0.75, the calculation based on Equation (14) yields an approximate $d_{tot}$ value of 1.578 μm. At this point, the focusing accuracy of the depth-sensing system is around 2/5 of the micro-objective’s depth of field.

## 6. Experimental Verification

**Figure 9:**
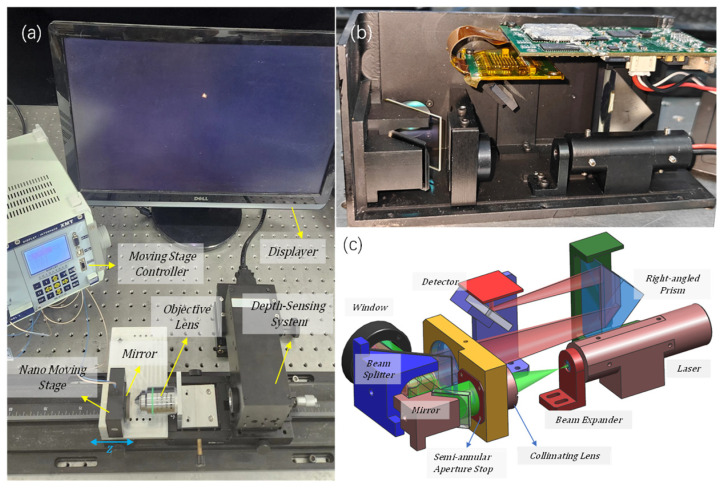
Schematic diagram of the experimental setup. (**a**) is the overall setup diagram of the experiment. (**b**) is the interior view of the depth-sensing system device. (**c**) is a simulation diagram of the parts of the depth-sensing system device.

To verify the accuracy of the optical simulation, an experimental test platform with the same parameters as those calculated in the simulation was constructed. As shown in Figure 9a, it includes a nano-displacement stage, a plane mirror (used to simulate the reflective surface of the samples), a Nikon Plan Apo 20×/0.75 objective, a semi-annular depth-sensing system, and an image acquisition system. Figure 9b,c show the internal optical path of the depth-sensing system. The light from the laser passes through the beam expander mirror and is expanded to match the size of the pupil of the microscope, which is 20 mm. Then, it passes through the semi-annular diaphragm plate with inner and outer diameters of 8 mm and 10 mm to produce the required semi-annular beam. Through the mirror, the semi-annular beam is reflected to the beam splitter mirror. Subsequently, it passes through the window and the microscope objective lens to reach the planar mirror. The reflected light passes through the microscope objective and window back to the beam splitter. The beam reflected by the beam splitter enters the imaging mirror. Since the focal length of the imaging mirror is 200 mm long, to avoid an overly long system, right-angled prisms are chosen to achieve the folding of the optical path. Then, through a small mirror, the reflected imaging beam is directed back to the detector of the imaging system. The CMV2000 model is chosen, and the image element size is 5.5 μm. By observing the shape of the focusing spot on the display (the more symmetrical the focusing spot, the better the coaxiality), the coaxial adjustment of the depth-sensing system, the microscope objective, and the plane reflector are achieved. The nano-displacement stage controls the positive and negative defocusing of the plane mirror in the direction of the optical axis, and the magnitude of defocusing is indicated by *z*.

**Figure 10:**
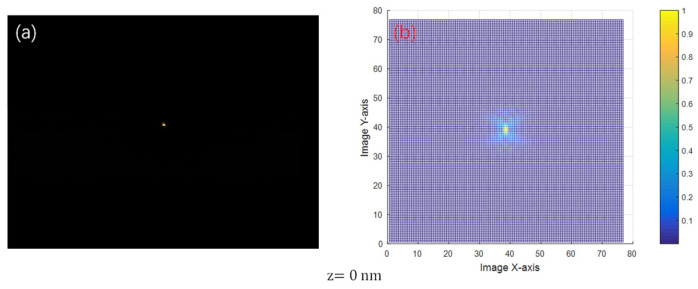
Spot diagram at *z* = 0 nm and its energy analysis. (**a**) is the spot at the focal point. (**b**) is an energy-analyzed three-dimensional diagram of the spot at the focal point.

The spot at *z* = 0 nm is taken for energy analysis. Its spot shape and energy distribution are shown in Figure 10. As can be seen from Figure 10b, the spot energy distribution is more concentrated, and all of it is concentrated at the main peak, which is the same as the optical simulation pattern. The zero point of the spot energy corresponds to the position of the dark ring. The first zero point of the spot’s *y*-axis in the figure is about 2.5 pixels away from the center. The detector pixel size is 5.5 μm. Thus, the radius of the first dark ring at the corresponding object surface is calculated to be approximately 0.688 μm. According to Rayleigh Criterion, the lateral resolution of the depth-sensing system is about 0.688 μm. The value of the interlayer resolution is calculated to be about 0.607 μm by Equation (4). Neglecting the error caused by optical hardware mounting, this result is consistent with the simulation results ($\delta_{r}$ = 0.68 μm, $\delta_{z}$ = 0.60 μm), which validates the correctness of the optical simulation.

**Figure 11:**
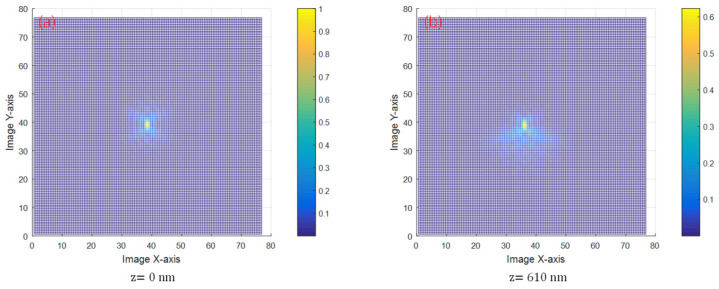
Plot of spot energy analysis at *z* = 0 nm and *z* = 610 nm. (**a**) is an energy-analyzed three-dimensional diagram of the spot at *z* = 0 nm. (**b**) is an energy-analyzed three-dimensional diagram of the spot at *z* = 610 nm.

When *z* increases to 610 nm, the spot changes are indiscernible to the naked eye. At this time, the spot energy analysis map is reprocessed and merged with the spot energy analysis map at z = 0 nm. The result is shown in Figure 11. As can be seen from Figure 11b, the center of spot energy at *z* = 610 nm is shifted from zero by approximately 0.68 μm, which is the length of the radius of the first dark ring of the *y*-axis of the Airy spot.

**Figure 12:**
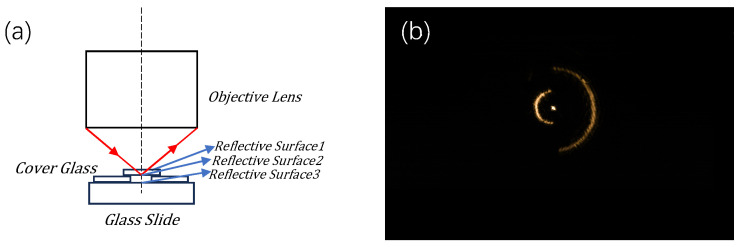
Schematic diagram of the three reflective surfaces and their spot maps. (**a**) is a schematic diagram of how the experimental setup is constructed. (**b**) is a graph of the experimental results obtained.

The three reflective surfaces of the sample (with the distances between the reflective surfaces all being greater than the interlayer resolution) were simulated by stacking one coverslip on top of two coverslips. The default objective lens was focused on the position of the second reflective surface. At this time, the schematic of the reflective surfaces and the corresponding spot conditions are shown in Figure 12.

## 7. Conclusions

This paper introduces a novel semi-annular aperture diaphragm depth-sensing microscope system as a response to the limitations faced by current autofocus systems. This system is designed to achieve real-time focusing on multiple reflective surfaces of transparent samples, enhancing focusing precision. Furthermore, a new formula for calculating the post-diffraction energy distribution resulting from a shaped aperture diaphragm is presented in this study. This formula accurately determines the size of the Airy spot on the focal plane.

Through experimental verification, the focusing accuracy of this proposed system can achieve 0.60 μm, surpassing the resolution capability of traditional active autofocus microscope systems. The enhanced resolving power reaches two-fifths of the microscope’s depth of field, making it suitable for high-resolution applications in industrial sectors such as DNA sequencing, bioengineering, and semiconductor automation.

## Figures and Tables

**Table 1:** Coordinate values of *X*-axis and *Y*-axis first amplitude zeros and system signal-to-noise ratio for spot with different *τ* values at *b* = 10 mm.

	$X_{0}$	$Y_{0}$	Signal-to-Noise Ratio
*τ* = 0.0	0.501 μm	1.142 μm	21.026 db
*τ* = 0.1	0.488 μm	1.125 μm	18.828 db
*τ* = 0.2	0.463 μm	1.118 μm	14.267 db
*τ* = 0.3	0.427 μm	0.902 μm	10.204 db
*τ* = 0.4	0.394 μm	0.862 μm	7.430 db
*τ* = 0.5	0.377 μm	0.853 μm	5.741 db
*τ* = 0.6	0.339 μm	0.761 μm	4.746 db
*τ* = 0.7	0.315 μm	0.695 μm	4.121 db
*τ* = 0.8	0.280 μm	0.680 μm	3.840 db
*τ* = 0.9	0.263 μm	0.637 μm	3.684 db

**Table 2:** Coordinate values of *X*-axis and *Y*-axis first amplitude zeros and system signal-to-noise ratio for spot with different *τ* values at *b* = 5 mm.

	$X_{0}$	$Y_{0}$	Signal-to-Noise Ratio
*τ* = 0.0	0.785 μm	1.645 μm	21.066 db
*τ* = 0.1	0.776 μm	1.622 μm	18.842 db
*τ* = 0.2	0.755 μm	1.620 μm	14.306 db
*τ* = 0.3	0.726 μm	1.586 μm	10.333 db
*τ* = 0.4	0.692 μm	1.571 μm	7.348 db
*τ* = 0.5	0.658 μm	1.570 μm	5.751 db
*τ* = 0.6	0.623 μm	1.501 μm	4.757 db
*τ* = 0.7	0.591 μm	1.430 μm	4.183 db
*τ* = 0.8	0.558 μm	1.359 μm	3.869 db
*τ* = 0.9	0.526 μm	1.290 μm	3.708 db

**Table 3:** Coordinate values of *X*-axis and *Y*-axis first amplitude zeros and system signal-to-noise ratio for spot with different *τ* values at *b* = 15 mm.

	$X_{0}$	$Y_{0}$	Signal-to-Noise Ratio
*τ* = 0.0	0.261 μm	0.547 μm	21.166 db
*τ* = 0.1	0.259 μm	0.541 μm	18.827 db
*τ* = 0.2	0.252 μm	0.540 μm	14.244 db
*τ* = 0.3	0.242 μm	0.529 μm	10.162 db
*τ* = 0.4	0.231 μm	0.523 μm	7.420 db
*τ* = 0.5	0.220 μm	0.522 μm	5.729 db
*τ* = 0.6	0.208 μm	0.500 μm	4.726 db
*τ* = 0.7	0.198 μm	0.475 μm	4.146 db
*τ* = 0.8	0.187 μm	0.452 μm	3.847 db
*τ* = 0.9	0.178 μm	0.430 μm	3.669 db

## Data Availability

Data are contained within the article.
